# The Truth of Unusual Deaths under Military Expansion: Evidence from the Stable Isotopes of a Human Skull Ditch in the Capital City of the Early Shang Dynasty

**DOI:** 10.3390/genes13112077

**Published:** 2022-11-09

**Authors:** Fang Fang, Jingwen Liao, Xiaomin Zeng, Juzhong Zhang

**Affiliations:** 1 Department for the History of Science and Scientific Archaeology, University of Science and Technology of China, Hefei 230026, China; 2 Department of Archaeological Sciences, Faculty of Archaeology, Leiden University, 2333 CC Leiden, The Netherlands; 3 Henan Provincial Institute of Cultural Heritage and Archaeology, Zhengzhou 450004, China

**Keywords:** Zhengzhou Shang City, human skull, isotope analysis, prisoner of war, Shang Dynasty, violence and warfare, biological archaeology

## Abstract

The site of Zhengzhou Shang City (ca. 1509-1315 cal. BC) was the capital of the early Shang Dynasty in China. Archaeological excavations have unearthed a ditch containing approximately one hundred unusual dead human skulls in the rammed-earth foundations of the palace area. The identity and origin of the skulls have long been disputed. In this work, strontium, carbon and nitrogen stable isotope analyses were carried out on 11 human skulls and 1 tooth from the ditch, as well as on 11 human bones, 11 human teeth from the ordinary tombs and 10 pig teeth from the Zhengzhou Shang City site. We determined that, in Zhengzhou Shang City, the local strontium isotope ratio ranges from 0.711606 to 0.711884, and ordinary inhabitants consumed mainly C_4_ plants supplemented by C_3_ plants. Moreover, humans buried in the ditch have ^87^Sr/^86^Sr values from 0.711335 to 0.711741 and consumed only C_4_ plants. Combining the isotopic data with the archaeological and cultural context, it is concluded that the unusual human skulls in the ditch are most likely those of prisoners of war captured by the central forces conquering the Xiaomintun area of Anyang in the early Shang Dynasty. The results provide valuable insight into the history of violence and military warfare in the early Chinese dynasty.

## 1. Introduction

Humans claim to have a history of civilization spanning more than 5000 years [1], and conflict and bloodshed caused by the human plundering of resources and the confrontation of regimes have filled the entire past [2,3,4,5]. Even today, civilization and violence still go hand in hand. Scalping and making skull cups were prevalent in past Eurasian societies as a form of violence, explained by archaeologists to be a ritual mortuary practice in the context of cannibalism and increased inter-group struggle [6,7,8,9,10,11]. In China, not only do historical documents record that ancient people collected the heads of enemies and made containers for trophies and revenge [12], but archaeological excavations have also revealed a surprising number of remains as a result of bloody killings in the prehistoric and Bronze Age, with the products of such violence found at the Zhengzhou Shang City and Dasima sites in Henan, the Jiangou site in Hebei and the Bianjiashan site in Zhejiang [13,14,15,16]. Although it is difficult today to reconstruct the entire process of past violence, archaeological sciences have the potential to present some new information.

**Figure 1:**
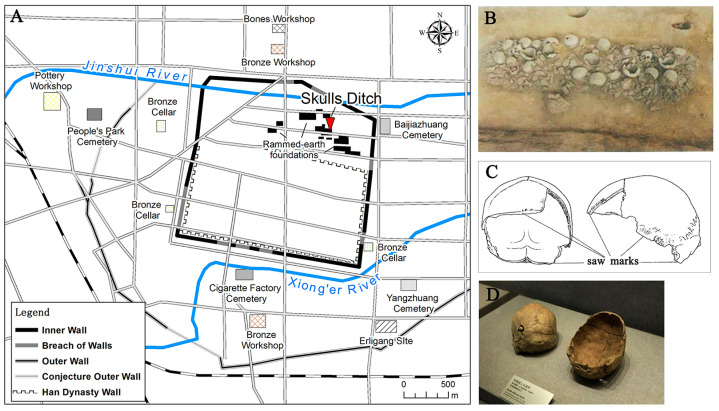
(**A**) The layout of Zhengzhou Shang City; (**B**) The human skull ditch; (**C**) Skulls with saw marks; (**D**) Human skulls on display at Henan Provincial Museum.

The Zhengzhou Shang City site (34°45′ N,113°41′ E, ca. 1509-1315 cal. BC, Figure 1A) is located in modern-day Zhengzhou city, Henan Province, China [17]. Since the 1950s, large-scale inner and outer city walls, palaces, water supply and drainage facilities, handicraft workshops, bronze cellars, sacrificial relics and tombs have been discovered here [18]. Its complex structures and functions have proved to be an early Shang Dynasty capital, with cultural findings predominantly representing the Erligang period (*二里冈*) [18,19,20], refreshing our understanding of early Chinese history. In 1974, a 15 m-long ditch filled with nearly 100 unusual dead human skulls was found during an excavation in the palace area of Zhengzhou Shang City (Figure 1A,B) [13,18]. According to the stratigraphy, the human skull ditch (HSD) dates to the first phase of the Erligang upper layer period (ca. 1427-1380 cal. BC) [17]. The majority of these human skulls were sawed transversely from the upper part of the ear and below the brow bone, leaving only the cranial portion (Figure 1C,D).

The excavators believe that the HSD is made up of discarded scraps from the slave owners of the Shang Dynasty, after sawing the skulls of slaves to make utensils [18]. This conclusion is a product of the era of the last century, with a focus on class struggle. Another scholar denied this view and compared the human sacrificial relics in the Anyang Yinxu site (late Shang Dynasty capital), judging that the identities of the human skulls in the Zhengzhou Shang City were prisoners of war [12]. Physical anthropological analysis indicates that individuals in the HSD ranged in age from 12 to over 40 years old, with the majority of adults being male, although the sex of some juveniles could not be determined [21]. In addition, evidence from the bones and teeth found in the HSD suggests that their owners generally suffered from malnourishment and anemia, showing poor health levels relative to those excavated from local common tombs [21]. The results seem to support the point that people found in the HSD were low-status slaves. However, it must be noted that, as the political center of the Shang Dynasty during its strong period, the material conditions of the inhabitants of Shang City were higher than those of other settlements; therefore, it was normal for Zhengzhou Shang City residents to have had a better standard of living than outsiders. In brief, the identity and origin of the HSD humans are still unknown with the current information.

In fact, there is no absolute gap between the identities of slaves and prisoners of war. What both have in common is that the main source of slaves was acquired through warfare with typical slave states, such as ancient Greece and Rome [22,23,24]. In China, a significant number of human sacrifice remains have been unearthed, dating from prehistory to early empires [25,26,27,28], although there is still controversy over whether the Xia, Shang and Zhou Dynasties were slave societies [25,29,30,31,32]. Previous research has extensively discussed the identity of these unnatural deaths in terms of historical documents and burial contexts, and it is generally recognized that these individuals had various social statuses as wives, concubines, courtiers, servants and slaves [33,34]. The establishment of the Shang and Zhou Dynasties was achieved by large-scale inter-communal wars [35]. In particular, by the upper Erligang period, the Shang Dynasty’s external military activities had expanded strongly to the surrounding area. The sphere of influence reached as far as Hebei in the north, Shandong in the east and the Jianghan region in the south, forming a wide kingship country area, with the Central Plains as the core region [36,37,38,39]. The defeated soldiers were captured and became slaves of the victors; this movable resource became one of the most significant natural resources of Shang society [40].

As mentioned above, and given the lack of written records from the early Shang Dynasty, it is hard to distinguish slaves from captives by archaeological contexts, but technological approaches can provide us with a window. Isotope analysis has proven that some of the human sacrifices at the Yinxu site, the capital of the late Shang Dynasty in Anyang, were captives taken by the Shang from outside the capital, especially the northwest area, and some were Qiang people [41], which also corroborates the Oracle Bone Inscriptions’ words about the Qiang people killed by the Shang people [42,43,44]. As the value of slaves is unpaid labor for slave owners, a logical assumption is that, if a soldier was killed soon after being captured or was sacrificed after a very short period of forced labor, they certainly could not be called a slave. Hence, we believe that whether one lived for a long time and engaged in productive activities in Zhengzhou Shang City can be used as a specific criterion for judging one as a slave or prisoner of war.

**Figure 2:**
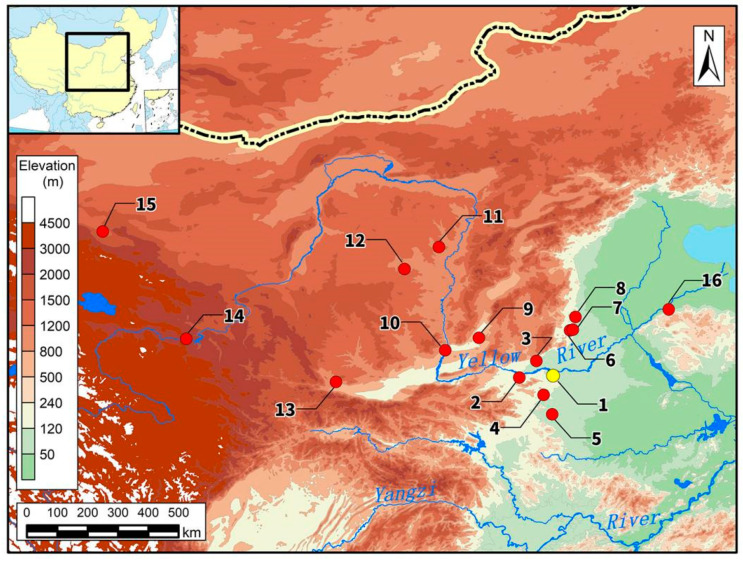
Location of Zhengzhou Shang City and other related archaeological sites. (1) Zhengzhou Shang City; (2) Erlitou; (3) Fucheng; (4) Wadian; (5) Jiahu; (6) Zhangdeng; (7) Xiaomintun; (8) Nancheng; (9) Taosi; (10) Ruiguo; (11) Shimao; (12) Jiadamao; (13) Xuechi; (14) Lajia; (15) Heishuiguo; and (16) Daxinzhuang.

In this work, to solve the long-standing mystery of the identity of the human skulls in the ditch of Zhengzhou Shang City, human bones and teeth from the HSD and ordinary tombs as well as pig teeth from Zhengzhou Shang City were collected for strontium, carbon and nitrogen isotope analyses (Supplementary Table S1). Moreover, benefiting from a series of related works on stable isotope studies carried out in China in recent years, we gathered the ranges of local strontium isotope ratios from 15 currently known sites within the sphere of influence of the Shang Dynasty as comparative material (Figure 2, Supplementary Table S2). The sites included the Nancheng cemetery [45], Ruiguo cemetery [46], Taosi site [47], Erlitou site [48], Wadian site [49], Jiahu site [50], Zhangdeng site [51], Xiaomintun site [52], Shimao site [53], Lajia Site [54], Heshuiguo site [55], Jiadamao site [56], Xuechi site [57], Fucheng site [51] and Daxinzhuang site [51].

## 2. Materials and Methods

Research has shown that domesticated pigs excavated locally are the preferred specimen for establishing local strontium isotope ratio ranges [58]. The origin of the pig as a domestic animal in China can be traced back to 9000 years ago [59,60], when it was already one of the main family of animals kept in the Shang Dynasty. Here, ten pig teeth unearthed from Zhengzhou Shang City were selected for strontium value testing. Additionally, 13 individuals from ordinary tombs at the Zhengzhou Shang City site were collected for carbon and nitrogen isotope analyses (including 11 human bones and 11 human teeth, of which 18 bones and teeth belonged to 9 identical individuals) to obtain dietary information for the residents in this area and to compare it with that of samples from the HSD. Judging by the specification of the graves, these 13 individuals were common inhabitants of the Zhengzhou Shang City site, and their activity dates are the same as those of the HSD samples [18]. For the focus of this study, we randomly sampled 11 human bones (strontium, carbon and nitrogen isotope analysis) and 1 human tooth (strontium isotope analysis) from the HSD. Detailed sampling data can be found in Supplementary Table S1. Our stable isotope experimental procedures followed the sampling protocol of previous studies [61,62,63].

The strontium isotope experiment was conducted at the Key Laboratory of Crust-Mantle Materials and Environments, University of Science and Technology of China. The enamel and compact bone were cut as test specimens after cleaning and polishing to remove soil and other contaminants. Afterwards, ultrasonic cleaning was performed three times with Milli-Q water for 30 min each. Then, 5% CH_3_COOH ultrasonic cleaning was conducted for 30 min, and the samples were left to stand for 8 h, before the acid was washed off Milli-Q water. The samples were placed in the muffle furnace and were ashed at 720 °C for 8 h. The ashed powder samples were weighed out as 0.1 g each and were stored in a low-pressure airtight dissolution cup. After dissolving the samples with 3 M HNO3, the strontium was collected through ion exchange chromatography on columns loaded with Sr-spec resin. Strontium isotope ratio measurements were conducted on the MAT262 Thermal ionization mass spectrometer (Finnigan Corp., San Jose, CA, USA) and were normalized to the NBS987 standard.

The carbon and nitrogen isotope experiments were conducted at the Laboratory of archaeological sciences, Peking University, China. Contaminants were first removed from the surfaces of the bones, and the samples were ground to powder. The samples were decalcified by adding 10% HCl for 3 days and were subsequently washed with 0.125 mol/L NaOH for 24 h to remove humic acid contamination. Afterward, 6% HCl was added to hydrolyze the samples at 70 °C, which were then dried and frozen to obtain gelatinized collagen. The carbon and nitrogen contents and isotope ratios of bone collagen samples were measured by an elemental analyzer Isotope Ratio Mass Spectrometer (EA-IRMS).

## 3. Results

The test results for 34 human tooth enamel and bone samples from 25 individuals and 10 pig tooth enamel samples excavated from Zhengzhou Shang City are listed in Supplementary Table S1. All specimens had C:N ratios of 3.2 and 3.4 in bone collagen, which is consistent with the characteristics of uncontaminated bone, and thus they can be considered qualified samples [64].

### 3.1. Radiocarbon Dating

To determine the date of HSD, one human skull fragment from Zhengzhou Shang City was sent to beta Analytic Inc., Miami, FL, USA, for radiocarbon analysis. The dates were calibrated using the Calib Radiocarbon Calibration Program (http://calib.org/calib/, accessed on 23 April 2018) and IntCal 13.14c [65]. Lab code 001 is dated to 1414-1260 cal. BC with 99.0% confidence (Table 1), which is in the first phase of the Erligang upper layer period.

### 3.2. Results of ^87^Sr/^86^Sr

**Figure 3:**
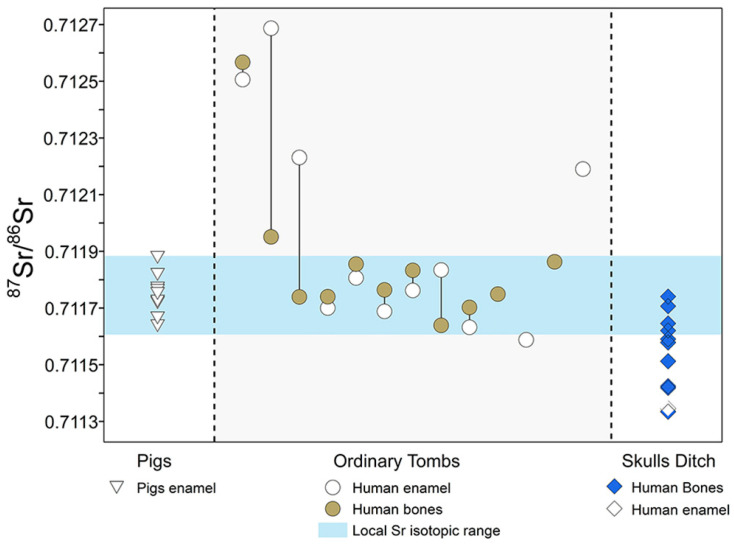
Human and animal ^87^Sr/^86^Sr values from Zhengzhou Shang City (the black line connecting the human enamel and bone samples represents the same individual).

The strontium isotope ratios of the ten pig enamels are close, with a mean value of 0.711745 and a two-fold standard deviation of 0.000139 (Figure 3). The local strontium isotope ratios should be 0.711606–0.711884.

The six tooth enamel samples of the 13 human individuals from the ordinary tombs have local ^87^Sr/^86^Sr values, and those of four enamel samples are above this, with a mean value of 0.711859. The ratio of the remaining one enamel sample is 0.711588, which is below the local benchmark (Figure 3). In addition, the Sr isotope ratios of nine individual bones are in the scope of the local range, whereas those of the other two bone samples are higher than the local reference value. The results of strontium isotope analysis indicate that migrating residents existed in Zhengzhou Shang City, which means that their birthplaces (lab ID: 11, 13, 15, 30 and 32) or settlements (lab ID: 12 and 14) were not in the capital. The most typical of these is the third sample of the ordinary tombs (lab ID: 15/16), where the individual was born in another area and then moved to Shang City for a long time. Therefore, the enamel value is outside the range, but that of bone collagen is inside the range.

A total of 12 individuals from the HSD have a mean strontium isotope ratio of 0.7115215, and the strontium isotope ratios range from 0.711335 to 0.711741, which is lower than the local ^87^Sr/^86^Sr range. The only tooth enamel sample collected from the HSD also reveals strontium values (=0.711346) that are different from that of Zhengzhou Shang City (Figure 3). Two individuals, numbered 39 and 44, have the same strontium isotope ratio, showing that these two persons likely came from the same region.

### 3.3. Results of δ^13^C and δ^15^N

**Figure 4:**
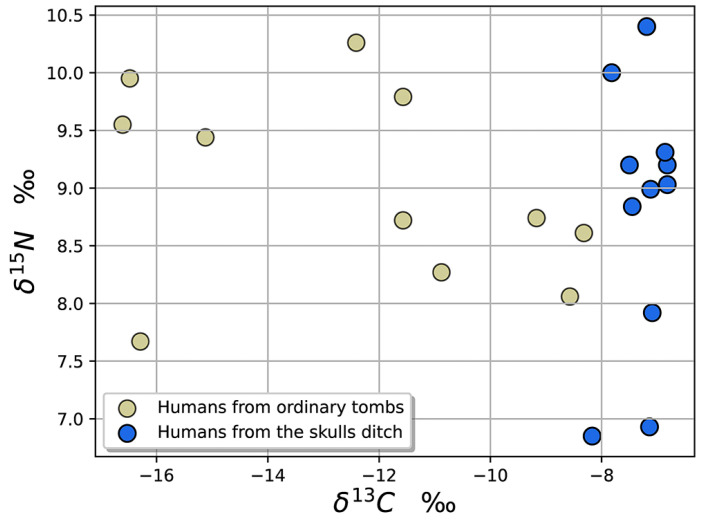
Human δ^13^C and δ^15^N values from Zhengzhou Shang City.

The δ^15^N values of the human bone samples in the ordinary tombs (*n* = 11) range from 7.67‰ to 10.26‰ (Figure 4). The δ^13^C values are distributed in the larger interval of −16.61‰ to −8.32‰ (Figure 4), suggesting that the plant food consumption of the common dwellers represented by these samples varied widely, with the majority of the diet consisting of C_4_ plants and the minority consisting of C_3_ plants.

The range of δ^13^C values for the human bones found in the HSD (*n* = 11) is −8.17‰ to −6.82‰ (Figure 4), indicating that their diet was dominated by C_4_-based food. The δ^15^N values range from 6.85‰ to 10.4‰ (Figure 4), which shows that the trophic level, in general, was at the same degree as the inhabitants of Zhengzhou Shang City.

## 4. Discussion

Multiple sources of isotope data reveal many differences between the humans of the HSD and those of the ordinary tombs at the Zhengzhou Shang City site. The ^87^Sr/^86^Sr range of HSD is 0.711335–0.711741, which is dissimilar to the local value. From the culinary information reflected by the C and N stable isotopes, the food composition of the buried humans was relatively rich, indicating a variety of dietary choices for the general population of the Zhengzhou Shang City, whereas the food of the humans in the HSD focused singularly on C_4_ plants. These differences suggest that those that experienced unusual deaths most likely came from other areas. In terms of meat intake levels, individuals in the HSD were neither inferior nor superior to the local general population in Zhengzhou Shang City. Integrating the overall data provided by the isotopes, it can be seen that the people found in the HSD did not live in Zhengzhou Shang City for a long time, and their nutritional level was close to that of the local ordinary residents of Shang City, which is not consistent with the identity of slaves. Therefore, we speculate that the people buried in the human skull ditch were residents who were from other areas where millet was the dominant food and who died and were buried uniformly in Zhengzhou Shang City for a certain reason.

**Figure 5:**
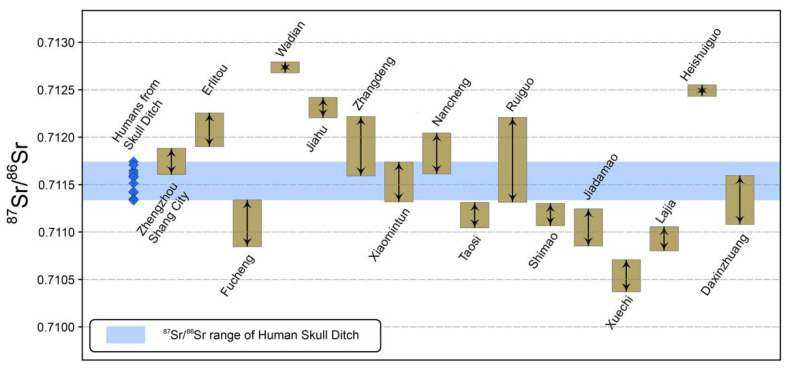
^87^Sr/^86^Sr values from the human skull ditch and the other 16 sites.

Further data (Figure 5) show that the strontium isotope ratio range of HSD closely matches that of the Xiaomintun site. Xiaomintun, located in modern Anyang city, Henan Province, China, is a site within the late Shang Dynasty capital (Yinxu site) [66], and its local strontium isotope ratio was determined by the nine pigs enamel samples, which range from 0.711319 to 0.711739, with a mean value of 0.711529 [52]. The ^87^Sr/^86^Sr values of all bones and teeth in the HSD are basically in this range, and only sample number 40 (=0.711741) deviates slightly but is very close to the upper limit of the interval. Furthermore, the dietary preferences of the HSD people were the same as those of the Yinxu area. Although there is no direct dietary evidence from the early to middle Shang Dynasty period of Yinxu, the available stable isotope and archaeobotanical data indicate that the crop structure of the Yinxu area in the late Shang Dynasty was dominated by millet, and the staple foods of the Yinxu people were all C_4_ plants [67,68,69]. Multiple shreds of evidence prove that the humans buried in the HSD at the Zhengzhou Shang City site had a high probability of coming from the Xiaomintun area of Yinxu, Anyang.

**Figure 6:**
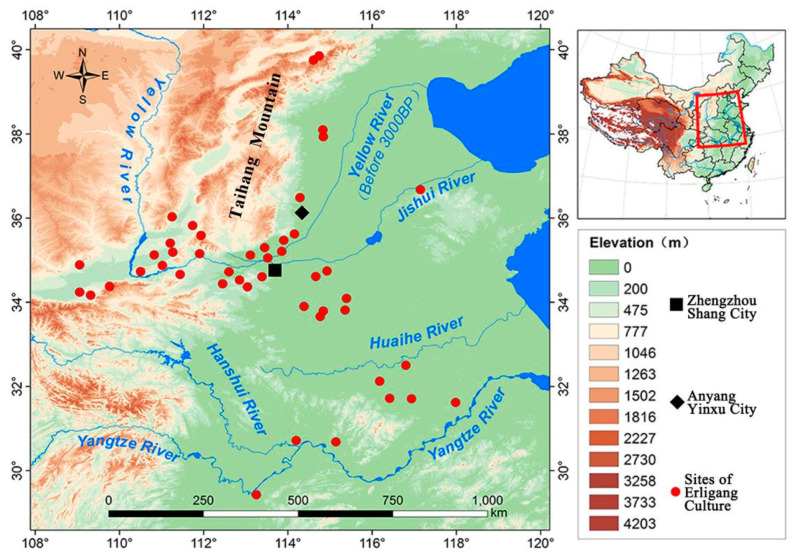
The location of sites of Erligang Culture and Yinxu City [51].

The HSD in Zhengzhou Shang City dates to the upper layer of the Erligang period (early Shang Dynasty). In contrast, the site of Anyang Yinxu City dates to a time later than the Erligang period. Although no remains of the early Shang culture have yet been discovered at Yinxu [70], important strongholds of the Erligang Culture existed on the north bank of the Zhanghe River north of Anyang and the Gaocheng area of Hebei farther north than the Zhanghe River (Figure 6) [71,72,73]. This indicates that the sphere of influence of the early Shang Dynasty expanded to central and southern Hebei. Anyang is situated on the main traffic route from Zhengzhou to the northern part of the North China Plain [74]. The early Shang Dynasty could not reach the Hebei without passing through the Anyang area.

Anyang is a plain region at the eastern foothill of the Taihang Mountain (Figure 6), which is suitable for agricultural production [75,76]. Additionally, there are important metal and salt resources distributed in the Shanxi and Hebei Provinces [77], which means that the conquest of Anyang allowed direct access to natural resources and further economic benefits. The Shang Dynasty in the Erligang period gradually entered a prosperous stage [78]. As a political, economic and cultural center, the highest-ranking social unit represented by Zhengzhou Shang City had the strength to conduct large-scale conquests in surrounding areas. Therefore, it was especially necessary for the early Shang rulers to open up a strategic route to the north and maintain the link between the central government and the north.

Before the Erligang period of the Shang Dynasty, Anyang was the distribution region of the Zhanghe-type Xiaqiyuan Culture (*漳河型下七垣*) [73,79]. In the late Erlitou Culture (generally considered to be the late Xia Dynasty period), the elites from the Xiaqiyuan Culture moved south and took control of the eastern part of the Central Plains, seizing control of the Erlitou capital in the Luoyang basin and beginning to build Zhengzhou Shang City as the capital of a widespread territorial state [80,81]. During this process, there might have been a small number of people left in the Anyang, or residents of other tribes may have moved in. By the time of the upper Erligang period, with the weakening of the Shang’s power in the Anyang region, this area was likely no longer under the control of the Shang Dynasty. Regardless, given that the Shang people moved their capital to Anyang thereafter [82,83,84], the central forces of the early Shang Dynasty launched military campaigns against the Anyang area to clear the way or to seize resources. The sawed-off skulls in the HSD of Zhengzhou Shang City site were likely those of prisoners of war acquired in the process. Moreover, the Shang Dynasty was able to reconquer this area due to a series of armed conflicts, allowing later Shang people to have unhindered access to Anyang and establishing their capital there for centuries.

## 5. Conclusions

In conclusion, the strontium, carbon and nitrogen stable isotope data, in conjunction with the stratigraphic and archaeological context of the remains, highly suggest that the people of the human skull ditch at Zhengzhou Shang City site were not slaves, but prisoners of war captured from the Xiaomintun area of Anyang. This study fills in the chronological gap between Zhengzhou Shang City and Anyang Yinxu Shang City to a certain extent and reveals a previously unknown bloody history between the two Shang capitals of China. Lastly, it should be noted that, although this research provides strong multidisciplinary evidence for the origin and identity of the individuals buried in the human skull ditch, the strontium isotope data do not cover the entire area where the early Shang Dynasty might have had a presence. We, therefore, acknowledge that more research is necessary to further verify the information on these unusual deaths.

## Figures and Tables

**Table 1:** Radiocarbon dating of a human skull fragment recovered from the Zhengzhou Shang City HSD.

Lab Code	Sample Type	Radiocarbon Age (^14^C yr BP)	cal. Years BC (1σ-Range)	Probability Distribution (%)	cal. Years BC (2σ-Range)	Probability Distribution (%)
001	Human skull	3070 ± 30	1392–1335	60.5	1414–1260	99.0
			1324–1288	39.5	1241–1235	1.0

## Data Availability

Not applicable.

## Supplementary Materials

The following supporting information can be downloaded at: https://www.mdpi.com/article/10.3390/genes13112077/s1. Supplementary Table S1: Detailed data of isotope experiment in Zhengzhou Shang City. Supplementary Table S2: Local strontium isotope ratio ranges for 15 relevant sites.
